# Cost-Effectiveness Analysis of Canagliflozin 300 mg Versus Dapagliflozin 10 mg Added to Metformin in Patients with Type 2 Diabetes in the United States

**DOI:** 10.1007/s13300-018-0371-y

**Published:** 2018-02-06

**Authors:** Cheryl Neslusan, Anna Teschemaker, Michael Willis, Pierre Johansen, Lien Vo

**Affiliations:** 1 Janssen Global Services, LLC, Raritan, NJ USA; 2 The Swedish Institute for Health Economics, Lund, Sweden; 3 Janssen Scientific Affairs, LLC, Titusville, NJ USA

**Keywords:** Canagliflozin, Cost-effectiveness, Dapagliflozin, Health economics, Modeling, QALY, Sodium glucose co-transporter 2 inhibitor, Type 2 diabetes mellitus

## Abstract

**Introduction:**

Agents that inhibit sodium glucose co-transporter 2 (SGLT2), including canagliflozin and dapagliflozin, are approved in the United States for the treatment of adults with type 2 diabetes mellitus (T2DM). SGLT2 inhibition lowers blood glucose by increasing urinary glucose excretion, which leads to a mild osmotic diuresis and a net loss of calories that are associated with reductions in body weight and blood pressure. This analysis evaluated the cost-effectiveness of canagliflozin 300 mg versus dapagliflozin 10 mg in patients with T2DM inadequately controlled with metformin in the United States.

**Methods:**

A 30-year cost-effectiveness analysis was performed using the validated Economic and Health Outcomes Model of T2DM (ECHO-T2DM) from the perspective of the third-party health care system in the United States. Patient demographics, biomarker values, and treatment effects for the ECHO-T2DM model were sourced primarily from a network meta-analysis (NMA) that included studies of canagliflozin and dapagliflozin in patients with T2DM on background metformin. Costs were derived from sources specific to the United States. Outcomes and costs were discounted at 3%. Sensitivity analyses that varied key model parameters were conducted.

**Results:**

Canagliflozin 300 mg dominated dapagliflozin 10 mg as an add-on to metformin over 30 years, with an estimated cost offset of $13,991 and a quality-adjusted life-year gain of 0.08 versus dapagliflozin 10 mg. Results were driven by the better HbA1c lowering achieved with canagliflozin, which translated to less need for insulin rescue therapy. Findings from sensitivity analyses were consistent with the base case.

**Conclusion:**

These results suggest that canagliflozin 300 mg is likely to provide better health outcomes at a lower overall cost than dapagliflozin 10 mg in patients with T2DM inadequately controlled with metformin from the perspective of the United States health care system.

**Funding:**

Janssen Scientific Affairs, LLC and Janssen Global Services, LLC.

**Electronic supplementary material:**

The online version of this article (10.1007/s13300-018-0371-y) contains supplementary material, which is available to authorized users.

## Introduction

Approximately 30 million people have diabetes in the United States [1]. The majority of people with diabetes (~ 90%) are classified as having type 2 diabetes mellitus (T2DM), which is characterized by insulin resistance, beta-cell dysfunction, and hyperglycemia [2, 3]. Chronic uncontrolled hyperglycemia in people with T2DM is associated with an increased risk of debilitating and potentially life-threatening micro- and macrovascular complications, including myocardial infarction (MI), stroke, end-stage renal disease (ESRD), and blindness, as well as premature death [4, 5]. Consequently, the economic burden of diabetes in the United States is staggering; in 2012, the estimated cost of diagnosed diabetes was $245 billion, of which 18% was attributed to prescription medications to treat complications of diabetes and 12% was attributed to the costs of anti-hyperglycemic agents (AHAs) and diabetes-testing supplies [6].

Controlling hyperglycemia can decrease the risk of complications in people with T2DM [5]. The American Diabetes Association (ADA) encourages a patient-centered approach to T2DM management, with consideration of the overall risk profile (i.e., beyond glycemic control) when identifying treatment targets [6]. Lifestyle modifications that result in healthier eating habits and increased physical activity are an important component of T2DM management that can lead to weight loss and improved glycemic control [7]. When pharmacologic intervention is needed, metformin is generally preferred; however, most patients will require a second medication soon after metformin is initiated in order to meet treatment targets [7]. Selection of a second-line therapy is at the discretion of the treating physician, with consideration of patient preferences, health history, and risk of side effects (e.g., hypoglycemia). Appropriate choices for combination therapy with metformin include sulfonylurea, thiazolidinedione, dipeptidyl peptidase-4 (DPP-4) inhibitors, sodium glucose co-transporter 2 (SGLT2) inhibitors, glucagon-like peptide-1 (GLP-1) receptor agonists, and basal insulin [6]. However, with emerging data demonstrating the cardioprotective effects of newer AHA classes (i.e., GLP-1 receptor agonists and SGLT2 inhibitors), some diabetes management guidelines have been revised to preferentially recommend the initiation of AHAs with demonstrated cardioprotective benefits [8].

Agents that inhibit SGLT2 are the newest class of AHAs approved for the treatment of T2DM. These drugs work to lower glucose by reducing the renal threshold for glucose (RT_G_), which increases urinary glucose excretion (UGE); SGLT2 inhibition is also associated with a mild osmotic diuresis and a net loss of calories that lead to blood pressure (BP) reduction and weight loss [9]. Because this mechanism of action works independently of insulin, SGLT2 inhibitors are complementary to other AHA classes (including insulin) and have an inherently low risk of hypoglycemia [9]. To date, four SGLT2 inhibitors—canagliflozin, dapagliflozin, empagliflozin, and ertugliflozin—have been approved for the treatment of T2DM in the United States, and the American Association of Clinical Endocrinologists (AACE) endorses the use of SGLT2 inhibitors as the first adjunctive oral AHA for combination therapy with metformin [8].

Canagliflozin and dapagliflozin were the first SGLT2 inhibitors approved in the United States. In phase 3 studies, canagliflozin and dapagliflozin improved glycemic control, lowered body weight, and reduced BP in patients with T2DM on a variety of background AHAs, including metformin, with greater improvements generally seen with the highest approved doses of each drug (canagliflozin 300 mg and dapagliflozin 10 mg) [10, 11]. There have not been any head-to-head clinical trials to evaluate the efficacy and safety of canagliflozin versus dapagliflozin in patients with T2DM, and, to date, the only head-to-head study of any SGLT2 inhibitors is a phase 1 study comparing the pharmacodynamic properties of canagliflozin 300 mg versus dapagliflozin 10 mg in healthy individuals [12]. In this study, canagliflozin 300 mg lowered RT_G_ to a greater extent than dapagliflozin 10 mg, resulting in ~ 25% more UGE over 24 h; in addition, canagliflozin 300 mg, but not dapagliflozin 10 mg, also delayed glucose reabsorption and decreased postprandial glucose [12]. Furthermore, unlike canagliflozin 100 mg and dapagliflozin 10 mg, canagliflozin 300 mg has been shown to reduce postprandial plasma glucose by transiently inhibiting intestinal SGLT1 [13]. In the absence of head-to-head clinical data in patients with T2DM, indirect results obtained using Bayesian network meta-analysis (NMA) have been used to compare the efficacy of canagliflozin and dapagliflozin as an add-on to metformin; the NMA results indicated that better HbA1c lowering was achieved with canagliflozin 300 mg versus dapagliflozin 10 mg [14–17]. The pharmacodynamic differences between canagliflozin and dapagliflozin may account for differences in efficacy and thus the differences in the occurrence of clinical outcomes with both agents.

Because T2DM is chronic and progressive, the financial burden over the long run is substantial. Efficient use of available health care resources requires economic assessment of the available treatment strategies, including estimation of cost-effectiveness, in order to inform the decision-making process [18]. Although drug acquisition costs are an important consideration, cost-effectiveness calculations must also capture the cost offsets and improved welfare that are associated with the better patient outcomes achieved with improved management of T2DM over time. Owing to the impracticality of obtaining evidence over a sufficient duration to capture the full impact of interventions over time, economic modeling is widely used as a method to generate such evidence, thus enabling assessment of the impact of alternative interventions [19, 20]. Economic simulations have been used to evaluate the cost-effectiveness of canagliflozin and dapagliflozin versus other AHA classes [21–28]. For example, canagliflozin 100 and 300 mg have demonstrated cost-effectiveness in second- and third-line therapy versus the DPP-4 inhibitor sitagliptin 100 mg in Mexico [21] and Canada [23]. Dapagliflozin has also been found to be cost-effective as monotherapy and in second-line therapy versus sulfonylurea, DPP-4 inhibitors, and acarbose in Nordic countries (i.e., Denmark, Finland, Norway, and Sweden) [22], the United Kingdom [24, 25], Greece [26], and China [27, 28]. There have been few reports of head-to-head cost-effectiveness comparisons of SGLT2 inhibitors [29–33]. Cost-effectiveness evaluations of canagliflozin versus dapagliflozin in the United Kingdom, Ireland, and Spain showed that canagliflozin was generally cost-effective compared with dapagliflozin as monotherapy and in second-line therapy with metformin [29–33]; however, similar analyses have not been conducted in the United States setting to the best of our knowledge.

As described above, the differential glucose-lowering efficacy seen for canagliflozin and dapagliflozin may impact health outcomes, making it suitable for comparison via economic simulations. The purpose of the analysis reported in the present paper was to compare the cost-effectiveness of canagliflozin 300 mg versus dapagliflozin 10 mg in patients with inadequate glycemic control on metformin monotherapy over 30 years from the perspective of the third-party payer in the United States health care system.

## Methods

### Model Description

**Fig. 1:**
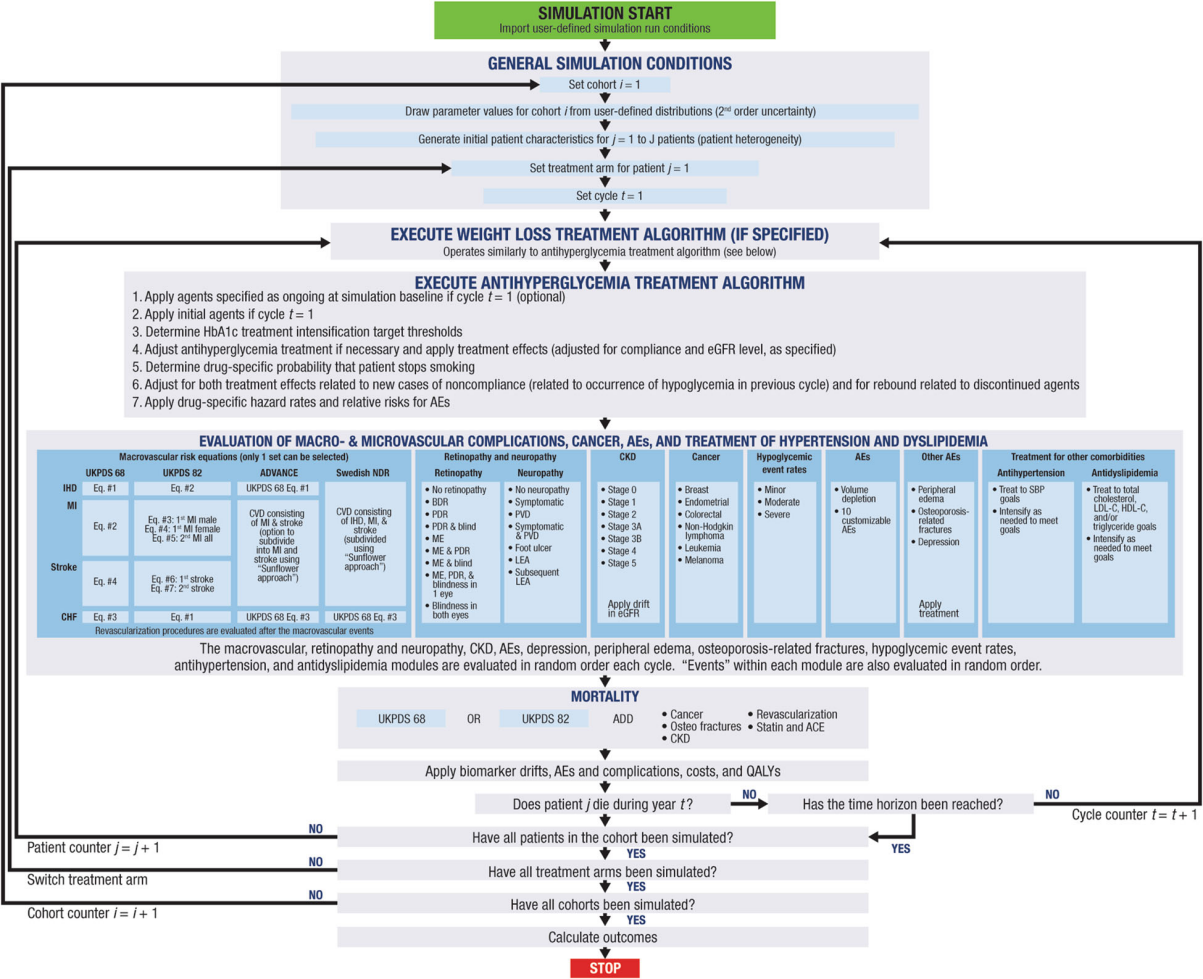
ECHO-T2DM model overview [34, 35]. *ACE* angiotensin-converting enzyme inhibitor, *AE* adverse event, *ADVANCE* Action in Diabetes and Vascular Disease: Preterax and Diamicron Modified-Release Controlled Evaluation, *BDR* background diabetic retinopathy, *CHF* congestive heart failure, *CKD* chronic kidney disease, *CVD* cardiovascular disease, *ECHO-T2DM* Economic and Health Outcomes Model of Type 2 Diabetes Mellitus, *eGFR* estimated glomerular filtration rate, *HDL-C* high-density lipoprotein cholesterol, *IHD* ischemic heart disease, *LDL-C* low-density lipoprotein cholesterol, *LEA* lower extremity amputation, *MI* myocardial infarction, *ME* macular edema, *NDR* National Diabetes Registry, *PDR* proliferative diabetic retinopathy, *PVD* peripheral vascular disease,* QALY* quality-adjusted life-year, *SBP* systolic blood pressure, *UKPDS* United Kingdom Prospective Diabetes Study. Reprinted from Neslusan et al. [21], copyright 2015, with permission from Elsevier

Simulations were performed using the Economic and Health Outcomes Model of T2DM (ECHO-T2DM), a stochastic, microsimulation (patient-level), cost-effectiveness model of T2DM treatment that is based on Markov health states that reflect the onset and progression of key micro- and macrovascular complications associated with T2DM (Fig. 1). The model has been validated for established effects of AHAs on parameters including glucose, body weight, BP, and renal function [19, 34, 35], and its application to cost-effectiveness evaluations of canagliflozin has been reported in detail elsewhere [21, 23].

The key simulation assumptions are summarized in Table 1. Briefly, the model randomly generates cohorts of individual hypothetical patients with T2DM based on patient characteristics from clinical trial data. Risk factor clustering of the key biomarkers [e.g., individuals who have higher body mass index (BMI) values tend to have less favorable cholesterol values] is supported [36]. Patients face risks for micro- and macrovascular complications and mortality using risk functions tailored to each patient’s characteristics. Transition probabilities for microvascular health states [e.g., retinopathy, chronic kidney disease (CKD), and neuropathy] are sourced from previous modeling studies and reflect differences in HbA1c levels and/or the duration of T2DM [37–40]. Event risks for macrovascular complications [e.g., MI, ischemic heart disease (IHD), congestive heart failure (CHF), and stroke] and mortality are modeled in this application using risk functions from the United Kingdom Prospective Diabetes Study (UKPDS 82) [41].

**Table 1:** Key simulation assumptions

Parameter	Assumption
Perspective	US third-party payer
Time horizon	30 years
Cycle length	1 year
Discount rate	3%
Treatment intensification threshold	
HbA1c	> 7.0%
SBP	> 140 mmHg
LDL-C	> 2.6 mmol/L (100 mg/dL)
Annual drift of biomarkers	
HbA1c (primary treatment)	0.14% [68]^a^
HbA1c (insulin rescue therapy)	0.15% [69]
SBP	0.3 mmHg [69]
Lipids	
Total cholesterol, LDL-C, HDL-C	0.008 mmol/L (0.03 mg/dL) [69]
Triglycerides	0.003 mmol/L (0.03 mg/dL) [69]
BMI	0 kg/m^2b^
Insulin rescue therapy treatment pathway	
1st rescue	Basal insulin (glargine) 10 IU/day, titrated up to 60 IU/day
2nd rescue	Prandial insulin (Humalog) 5 IU/day, titrated up to 200 IU/day
Anti-hypertension treatment algorithm	
1st treatment	ACE inhibitor (ramipril 8 mg)
2nd treatment	Diuretic (hydrochlorothiazide 25 mg)
3rd treatment	Calcium channel blocker (felodipine 7.5 mg)
4th treatment	Beta blocker (metoprolol 125 mg)
Anti-dyslipidemia treatment	Atorvastatin 10 mg → 20 mg → 40 mg → 80 mg
Macrovascular risk equations	UKPDS 82 [41]
Microvascular risk equations	CDC model of CKD, WESDR, REP [39, 40, 70, 71]

*ACE* angiotensin-converting enzyme, *BMI* body mass index, *CDC* Centers for Disease Control and Prevention, *CKD* chronic kidney disease, *HDL-C* high-density lipoprotein cholesterol, *LDL-C* low-density lipoprotein cholesterol, *REP* Rochester Epidemiology Project, *SBP* systolic blood pressure, *UKPDS* United Kingdom Prospective Diabetes Study, *WESDR* Wisconsin Epidemiologic Study of Diabetic Retinopathy

^a^Canagliflozin 300 mg and dapagliflozin 10 mg were assumed to have the same drift as the metformin arm from the 5-year ADOPT (A Diabetes Outcome Progression Trial) study

^b^Assumed to plateau (i.e., no drift)

An AHA treatment algorithm governs treatment intensification. Specifically, patient biomarkers deteriorate annually according to established “drifts” that reflect the progressive nature of the disease; these drifts are updated within each cycle [42, 43]. For example, when the drift causes patients to exceed HbA1c treatment target thresholds [and/or when adverse event (AE)-related treatment discontinuation occurs], the next agent in the treatment sequence is activated. Similar treatment algorithms for hypertension and dyslipidemia can be applied to intensify treatment when BP and lipid targets are not met.

ECHO-T2DM was used to model two types of hypoglycemic events in this analysis: nonsevere and severe. Severe hypoglycemic events included episodes that required assistance or resulted in seizure or loss of consciousness; nonsevere symptomatic hypoglycemic events included episodes in which symptoms were accompanied by a glucose reading ≤ 3.9 mmol/L (70 mg/dL) and did not meet the criteria for a severe episode. In the current analysis, AEs potentially related to the mechanism of SGLT2 inhibition (e.g., urinary tract infections [44], genital mycotic infections (e.g., yeast infections) [45], volume depletion-related events [46], and osmotic diuresis-related events [47]) were modeled. In this analysis, treatment with canagliflozin and dapagliflozin is discontinued when the simulated estimated glomerular filtration rate (eGFR) falls below 60 mL/min/1.73 m^2^, consistent with the prescribing information for both drugs [48, 49].

Costs are assigned for treatment interventions, complications, and AEs. Quality-adjusted life-year (QALY) disutility weights, which reflect decrements in quality of life that are associated with the negative impact of each health state, are applied for each patient during each cycle. To deflate costs and QALYs to their net present value, a discount rate of 3% is applied [50]. Outcomes include biomarker evolution curves, cumulative incidences, and relative risk reductions (RRRs) in micro- and macrovascular events, life-years and QALYs, and cost and cost-effectiveness metrics.

### Simulation Parameters and Patient Profile

In these analyses, 1000 cohorts of 2000 hypothetical patients (i.e., 2,000,000 unique patients) were generated and their health histories were simulated over 30 years. Patient characteristics were based on random draws from distributions of initial patient characteristics from a pooled analysis of data from two canagliflozin clinical trials that included patients who were on background therapy with metformin [51, 52] (Table S1 in the Electronic supplementary material, ESM).

### Treatment Effects and Algorithm

Two identical cohorts of hypothetical patients with T2DM inadequately controlled on metformin were assigned to canagliflozin 300 mg or dapagliflozin 10 mg, respectively. Treatment effects for canagliflozin 300 mg were sourced from a previously reported Bayesian NMA of 26-week data from clinical trials in patients with T2DM who were on background metformin (Table 2) [14, 17]. For parameters not available in the NMA (i.e., treatment effects for lipids, rates of hypoglycemia and AEs), pooled canagliflozin 300 mg data from the head-to-head studies of canagliflozin in dual therapy with metformin were assumed for the canagliflozin 300 mg arm; pooled canagliflozin 100 mg data from the same studies were conservatively assumed for the dapagliflozin 10 mg arm [51, 52].

**Table 2:** Treatment effects for canagliflozin 300 mg and dapagliflozin 10 mg^a^

Parameter	Canagliflozin 300 mg	Dapagliflozin 10 mg
Clinical parameters		
HbA1c, %	– 0.79	– 0.41
SBP, mmHg	– 5.4	– 3.7
BMI, kg/m^2^	– 0.8	– 0.7
Total cholesterol, mmol/L (mg/dL)	0.27 (10.4)	0.14 (5.5)
LDL-C, mmol/L (mg/dL)	0.21 (8.0)	0.13 (5.1)
HDL-C, mmol/L (mg/dL)	0.11 (4.4)	0.09 (3.6)
Triglycerides, mmol/L (mg/dL)	– 0.13 (– 11.9)	– 0.18 (– 16.1)
Hypoglycemia events, per patient-year of exposure		
Nonsevere symptomatic	0.050	0.050
Severe	0.001	0.001
AEs, per patient-year of exposure		
Male genital mycotic infection	0.067	0.068
Female genital mycotic infection	0.138	0.129
Lower UTI	0.079	0.087
Upper UTI	0.001	0.003
Volume depletion-related AEs	0.018	0.016
Osmotic diuresis-related AEs	0.038	0.056
Discontinuation rate associated with AEs in the first year, %	5.2	5.2

*AE* adverse event, *BMI* body mass index, *HDL-C* high-density lipoprotein cholesterol, *LDL-C* low-density lipoprotein cholesterol, *NMA* network meta-analysis, *SBP* systolic blood pressure, *UTI* urinary tract infection

^a^Data for treatment effects were sourced from a 26-week NMA of anti-hyperglycemic agents, which included canagliflozin and dapagliflozin, in dual therapy with metformin [17]; for parameters not available in the NMA (i.e., hypoglycemia, AE rates, lipids), data were assumed based on a pooled analysis of two canagliflozin trials [51, 52]

As described previously, treatment with canagliflozin 300 mg and dapagliflozin 10 mg was discontinued when eGFR was simulated to fall below 60 mL/min/1.73 m^2^. In the first year, patients discontinued canagliflozin 300 mg or dapagliflozin 10 mg according to the rates observed in the pooled analysis of the two head-to-head trials [51, 52]. Treatment was first intensified with basal insulin (glargine) starting at 10 IU/day and was titrated to a maximum dose of 60 IU/day during a cycle in which HbA1c exceeded 7.0%. Prandial insulin (Humalog) was added next at 5 IU/day and titrated up to 200 IU/day to maintain HbA1c < 7.0% in patients who needed additional glycemic control. Treatment effects and hypoglycemia incidences associated with the use of basal and prandial insulin were sourced from the literature [53–56]. Hypertension and lipid treatment algorithms were also applied based on meeting systolic BP (SBP) < 140 mmHg and low-density lipoprotein cholesterol ≤ 2.6 mmol/L (100 mg/dL).

### Cost and Utilities

Costs for diabetes complications were largely sourced from an analysis of medical costs in the United States [57] (Table S2 in the ESM). Costs related to CKD were obtained from the Centers for Disease Control and Prevention (CDC) model of CKD [39]. All costs were inflated to 2016 US dollars. Canagliflozin 300 mg was priced at $14.22 per day and dapagliflozin 10 mg was priced at $14.35 per day [58]. Patient preferences for health status were captured using published QALY disutility weights from studies of patients with T2DM, where possible [38, 59, 60]. In this analysis, a threshold of $100,000 per QALY gained was used to define whether an intervention can be considered cost-effective [61].

### Outcomes

Biomarker evolution curves were simulated over 30 years. Outcomes also included cumulative incidence of micro- and macrovascular complications and RRRs associated with each complication for canagliflozin versus dapagliflozin. Differences in the costs and QALYs were generated for canagliflozin versus dapagliflozin. Incremental cost-effectiveness ratios (ICERs), scatterplots of the cost-effectiveness planes, and cost-effectiveness acceptability curves were computed.

### Sensitivity Analyses

One-way sensitivity analyses were conducted to evaluate the robustness of the base case results by varying key model parameters. Alternative assumptions used in these analyses included:

- *Alternative treatment thresholds*: changing the HbA1c intensification threshold to 7.5% (SA1) or 8.0% (SA2).
- *Patient characteristics*: using patient characteristics from real-world data (Table S1 in the ESM; SA3).
- *Time horizon*: using alternative time horizons of 20 years (SA4) or 10 years (SA5).
- *Costs*: using a generic price for basal insulin ($0.21 per day vs $0.25 per day in the base case; SA6).

### Compliance with Ethics Guidelines

This article does not contain any new studies with human or animal subjects performed by any of the authors.

## Results

### Base Case

Over 30 years, canagliflozin 300 mg was associated with lower costs and larger improvements in QALYs compared with dapagliflozin 10 mg; therefore, canagliflozin 300 mg “dominated” dapagliflozin 10 mg (Table 3). Relative to dapagliflozin 10 mg, costs were $13,991 lower, life-years were 0.02 higher, and QALYs were 0.08 higher with canagliflozin 300 mg.

**Table 3:** Base case results: costs and QALYs over 30 years

	Canagliflozin 300 mg	Dapagliflozin 10 mg	Difference
Costs (discounted), $			
Macrovascular			
MI	11,500	11,667	– 166
IHD	4984	5029	– 44
CHF	3068	3108	– 40
Stroke	6965	7079	– 114
Microvascular			
Retinopathy	795	808	– 13
CKD	6595	6645	– 51
Neuropathy	3571	3593	– 22
AHA			
Oral agents	61,130	61,574	– 444
Insulin	69,135	82,159	– 13,023
Prescription treatment			
Hypoglycemia	177	209	– 31
AEs	243	264	– 21
Hypertension	323	385	– 62
Dyslipidemia	757	715	42
Total costs, $	169,244	183,235	– 13,991
Health indicators (discounted)			
LYs	14.33	14.31	0.02
QALYs	10.04	9.96	0.08
Survival, %^a^	23.7	23.6	0.1
Cost per QALY, $			Dominating

*AE* adverse event, *AHA* anti-hyperglycemic agent, *CHF* congestive heart failure, *CKD* chronic kidney disease, *IHD* ischemic heart disease, *LY* life-year, *MI* myocardial infarction, *QALY* quality-adjusted life-year

^a^Percentage alive at end of simulation

The cost offsets and QALY gains were primarily driven by better HbA1c lowering with canagliflozin 300 mg versus dapagliflozin 10 mg, although larger SBP reductions and greater weight loss were also contributors. Figure S1 in the ESM displays the simulated values of these biomarkers over time. Per the NMA results, the HbA1c reduction with canagliflozin 300 mg was greater than that with dapagliflozin 10 mg in the first year; HbA1c drifted upward over time and eventually converged near the target level of 7.0% for both treatments as patients were initiated on insulin with dose adjustments as needed to reach the target.

The improvements in these biomarkers translated into decreased event rates for most macrovascular complications (except peripheral vascular disease, which is not associated with HbA1c in ECHO-T2DM) with canagliflozin 300 mg versus dapagliflozin 10 mg. Event rates were also smaller for some microvascular complications, including background diabetic retinopathy, proliferative diabetic retinopathy, macular edema, blindness, symptomatic neuropathy, and microalbuminuria, with canagliflozin 300 mg versus dapagliflozin 10 mg (Table 4). Due to the better HbA1c-lowering efficacy, patients treated with canagliflozin 300 mg had less need for rescue therapy with insulin over time compared with patients treated with dapagliflozin 10 mg (Fig. S2 in the ESM). As a result of less insulin use, hypoglycemia event rates were lower with canagliflozin 300 mg versus dapagliflozin 10 mg. This decreased incidence of hypoglycemia episodes with the use of canagliflozin 300 mg versus dapagliflozin 10 mg was a key driver of the QALY gain. Increased survival and better weight outcomes were also notable contributors to the QALY gain with canagliflozin (Table S3 in the ESM).

**Table 4:** Cumulative incidences and RRRs of micro- and macrovascular events over 30 years

Cumulative incidence, %	Canagliflozin 300 mg	Dapagliflozin 10 mg	RRR
Macrovascular			
MI	19.1	19.4	1.5
IHD	18.1	18.3	1.0
CHF	12.0	12.1	1.0
Stroke	6.8	6.9	0.9
PVD	40.2	40.1	– 0.1
CHD (MI and IHD)	33.1	33.5	1.2
CVD (MI, IHD, and stroke)	37.8	38.2	1.0
Microvascular			
Background diabetic retinopathy	40.0	40.4	1.1
Proliferative diabetic retinopathy	1.6	1.6	2.1
Macular edema	23.4	23.8	1.6
Blindness			
One eye	1.4	1.5	1.8
Both eyes	1.3	1.3	1.9
Symptomatic neuropathy	29.9	29.9	0.1
Diabetic foot ulcer	24.8	24.8	0.0
Lower extremity amputation	14.9	14.9	0.2
Microalbuminuria	10.4	10.5	0.9
Macroalbuminuria	6.9	6.9	– 0.4
CKD stage 3a	35.5	35.4	– 0.2
CKD stage 3b	23.3	23.2	– 0.4
CKD stage 4	14.5	14.5	– 0.2
CKD stage 5	7.8	7.7	– 0.5
ESRD	6.7	6.7	– 0.5

*CHD* coronary heart disease, *CHF* congestive heart failure, *CKD* chronic kidney disease, *ESRD* end-stage renal disease, *IHD* ischemic heart disease, *MI* myocardial infarction, *PVD* peripheral vascular disease, *RRR* relative risk reduction

The clinical benefits of canagliflozin 300 mg versus dapagliflozin 10 mg translated into cost offsets. The largest cost offset was attributable to lower insulin acquisition costs ($13,023) because canagliflozin 300 mg delayed the need for insulin rescue therapy compared with dapagliflozin 10 mg (Table 3). Small cost offsets were seen for most other outcomes.

**Fig. 2:**
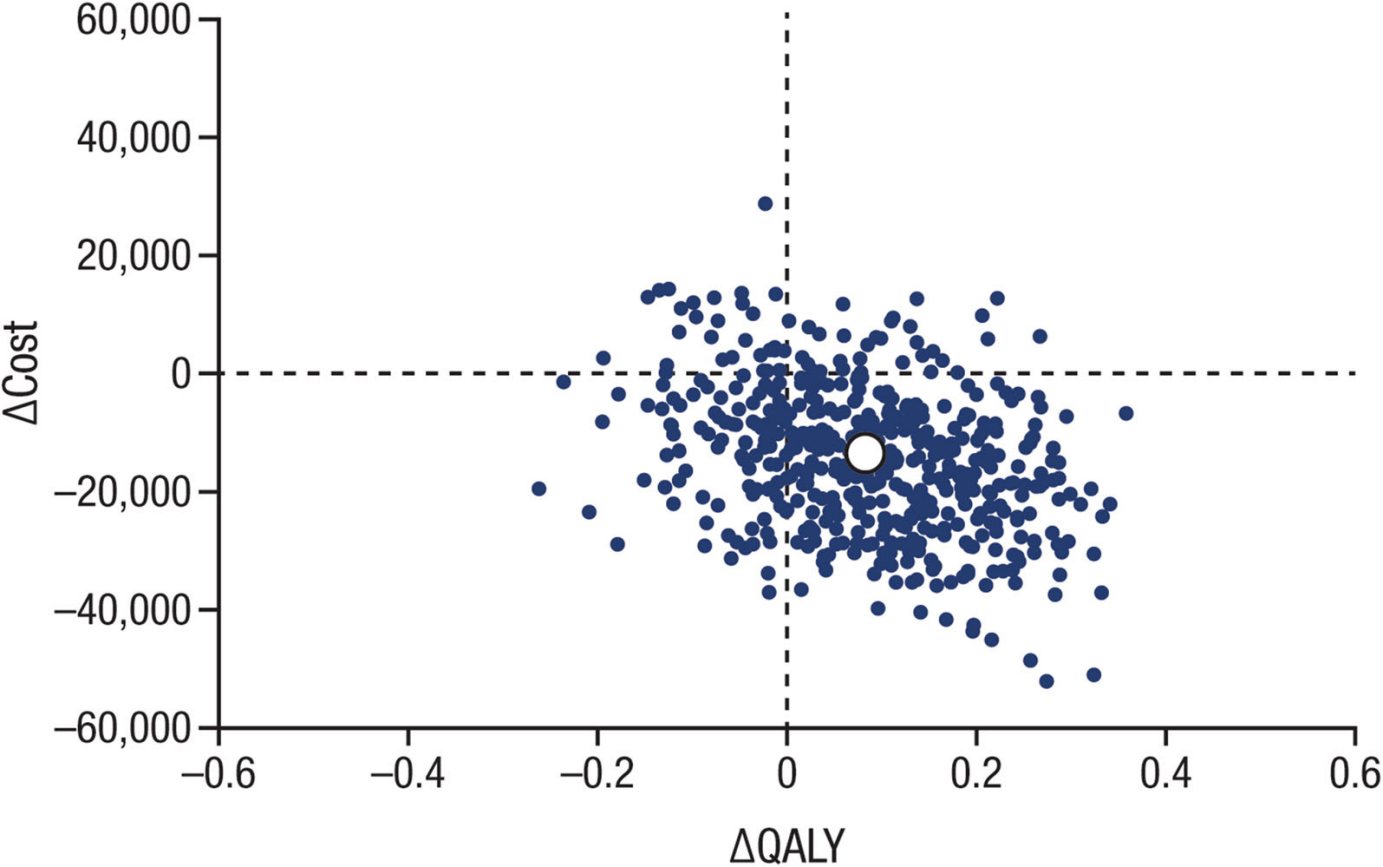
Cost-effectiveness plane for canagliflozin 300 mg versus dapagliflozin 10 mg. *QALY* quality-adjusted life-year. White symbol indicates the mean

**Fig. 3:**
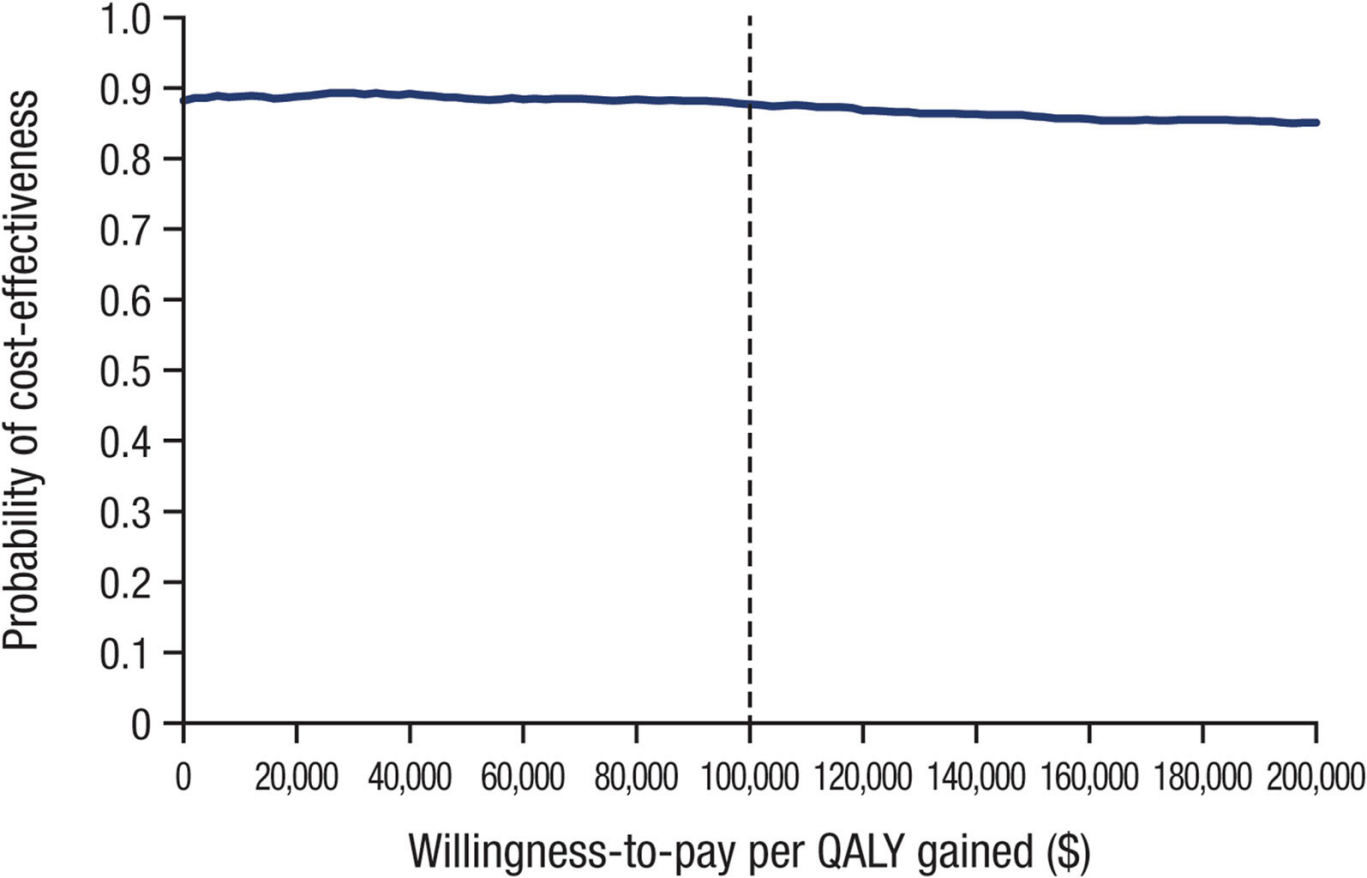
Cost-effectiveness acceptability curve for canagliflozin 300 mg versus dapagliflozin 10 mg. *QALY* quality-adjusted life-year

Canagliflozin 300 mg was associated with QALY gains and decreased costs in the majority (~ 69%) of cohort replications (southeast quadrant; Fig. 2). The likelihood is high that canagliflozin 300 mg is cost-effective versus dapagliflozin 10 mg across a wide range of willingness-to-pay values (see Fig. 3). Given that the majority of replicates were associated with lower total costs, the probability that canagliflozin 300 mg is cost-effective was 88% even at a willingness-to-pay level of $0 per QALY gained. Because so many replicates are in the southeast quadrant (and thus are invariant to changes in willingness to pay), the cost-effectiveness acceptability curve remains relatively unchanged over a range of willingness-to-pay thresholds up to $100,000 per QALY gained (i.e., the level used to define cost-effective in the United States [61]).

### Sensitivity Analyses

Consistent with the base case, canagliflozin 300 mg dominated dapagliflozin 10 mg in each of the alternative scenarios evaluated in the sensitivity analyses (Table 5). Cost offsets and QALY gains were generally similar to the base case when treatment intensification occurred at the HbA1c threshold of 7.5% (SA1) versus 7.0%; however, the cost offsets were slightly smaller when the treatment intensification threshold was 8.0% (SA2). The use of patient characteristics derived from a sample of patients in actual clinical practice instead of the clinical trials (SA3) yielded slightly smaller cost offsets but similar QALY gains to those in the base case. Simulating shorter time horizons of 20 years (SA4) and 10 years (SA5) resulted in slightly smaller cost offsets and QALY gains compared with the base case. As expected, cost offsets were smaller with canagliflozin 300 mg versus dapagliflozin 10 mg when a generic price was used for basal insulin (SA6).

**Table 5:** Sensitivity analysis results: canagliflozin 300 mg versus dapagliflozin 10 mg

Sensitivity analysis	ΔCost ($)	ΔQALY	ICER ($ per QALY gained)
Base case	– 13,991	0.08	Dominating
SA1: HbA1c treatment intensification at 7.5%	– 11,706	0.07	Dominating
SA2: HbA1c treatment intensification at 8.0%	– 8689	0.07	Dominating
SA3: real-world patient characteristics	– 11,786	0.07	Dominating
SA4: 20-year time horizon	– 11,646	0.07	Dominating
SA5: 10-year time horizon	– 6269	0.03	Dominating
SA6: generic price for basal insulin	– 12,974	0.08	Dominating

*AE* adverse event, *ICER* incremental cost-effectiveness ratio, *QALY* quality-adjusted life-year

## Discussion

Economic simulation results suggest that canagliflozin 300 mg dominated dapagliflozin 10 mg as add-on to metformin in the United States from the perspective of the third-party payer. QALY gains and lower costs were observed with canagliflozin versus dapagliflozin in the majority of cohort replications, indicating a reasonable level of certainty.

The improvements in health outcomes and cost offsets seen with canagliflozin were largely attributable to better control of key biomarkers, including HbA1c, weight, and SBP. Of particular importance was the better glycemic control with canagliflozin 300 mg compared with dapagliflozin 10 mg, which was modeled based on previously reported NMA results that were used in the absence of head-to-head clinical trial data [14, 17]. Note that other NMAs using different methodologies [14–16] have reported similar findings to the NMA utilized in this study. The previously reported pharmacodynamic data that demonstrated greater UGE with canagliflozin 300 mg versus dapagliflozin 10 mg [12] support the findings from these NMAs. The greater HbA1c lowering with canagliflozin was associated with a delay in intensification with insulin rescue, which not only led to greater QALY gains via fewer hypoglycemic events and avoided weight gain, but also yielded substantial cost offsets, primarily related to the acquisition costs of insulin.

Results from each sensitivity analysis corroborated the base case findings, as canagliflozin dominated dapagliflozin in scenarios that may be more reflective of the patient experience in the United States. Given the emphasis on patient-centered care, a higher HbA1c target may be appropriate in some patients; in the sensitivity analyses that used treatment intensification thresholds of 7.5% or 8.0%, canagliflozin continued to dominate dapagliflozin. With the assumption that the clinical effects from the trials would be transferable to a different patient population in a nonrandomized setting, canagliflozin also dominated dapagliflozin when patient characteristics from a real-world United States patient population were used. This suggests that canagliflozin 300 mg may be a cost-effective treatment option in actual clinical practice. Despite the chronic nature of T2DM, better health outcomes and lower costs of canagliflozin treatment were realized even over the shorter time horizons of 10 and 20 years, with canagliflozin dominating dapagliflozin. In addition, assuming the use of a lower-cost insulin treatment regimen as rescue therapy had no notable impact on the simulation results, as canagliflozin 300 mg continued to dominate dapagliflozin 10 mg.

Economic simulation modeling is a widely used tool (endorsed by the ADA Consensus Panel [20]) that can be used to generate evidence needed to help make informed decisions about clinical outcomes, costs, and QALYs of competing T2DM treatments. A limitation of the analysis is the lack of head-to-head clinical evidence for canagliflozin and dapagliflozin in patients with T2DM. Therefore, treatment effects were sourced from a previously reported Bayesian NMA of 26-week data from clinical trials that included studies of canagliflozin and dapagliflozin in combination with metformin [14, 17]. The NMA results used to inform these simulations were robust, and the greater efficacy of canagliflozin versus dapagliflozin used as inputs in the model was in line with observations from similar analyses [15, 16, 62]. Of note, the use of 26-week data for the NMA is supported by the clinical data for each individual drug, as the nadir in glycemic efficacy was observed near this time and was shown to be sustained for 52 weeks in longer studies [51, 52, 63]. For comparison, a NMA using 52-week data from add-on to metformin studies showed similar reductions in HbA1c (– 0.76% with canagliflozin 300 mg and – 0.48% with dapagliflozin 10 mg) with a considerably smaller network, and using these values as treatment effects in the cost-effectiveness simulations produced results consistent with the simulations based on the 26-week data (data not shown). Because an evidence-based approach was used to reduce bias in the model, conservative modeling assumptions were used for parameters that lacked reliable data sources or had relatively higher levels of uncertainty.

The model accounts for the potential impacts of AEs that are known to be related to the mechanism of SGLT2 inhibition. However, the comparative risk associated with canagliflozin and dapagliflozin treatment on amputation, which was identified as a safety risk in the CANagliflozin cardioVascular Assessment Study (CANVAS) Program [64], was not modeled owing to the lack of comparable data for dapagliflozin. Analysis of safety data from the Multicenter Trial to Evaluate the Effect of Dapagliflozin on the Incidence of Cardiovascular Events (DECLARE) will be important to confirm or refute whether amputation is a class effect [65], and emerging safety data from this study will be incorporated into future modeling exercises.

This analysis is strengthened by the use of ECHO-T2DM, which has demonstrated good model validity for established effects of AHAs on intermediate biomarkers such as glucose, body weight, BP, and renal function [34], but it does not yet account for the potential direct cardioprotective effects of SGLT2 inhibitors that have been reported recently [64, 66]. In the CANVAS Program, canagliflozin was associated with a reduced risk of cardiovascular death, nonfatal MI, or nonfatal stroke compared with placebo in patients with T2DM and established cardiovascular disease or cardiovascular risk factors [64]. Similar observations in patients with established cardiovascular disease from the Empagliflozin Cardiovascular Outcome Event Trial in Type 2 Diabetes Mellitus Patients (EMPA-REG OUTCOME) and the CVD-REAL observational study suggest that cardioprotection is likely to be a class effect for SGLT2 inhibitors [66, 67]. Cardiovascular outcomes data from the CANVAS Program and EMPA-REG OUTCOME, together with forthcoming data from DECLARE, will help to inform any potential SGLT2 inhibition-mediated cardioprotective effects in updates to the ECHO-T2DM model. Including assumptions regarding this anticipated class effect in future modeling exercises will be particularly important for comparisons with other AHA classes.

## Conclusion

This cost-effectiveness analysis suggests that canagliflozin 300 mg is likely to provide better clinical outcomes and more QALYs at a lower cost than dapagliflozin 10 mg in dual therapy for T2DM patients with a background of metformin from the perspective of the third-party payer in the United States health care system. These findings suggest that treatment with canagliflozin versus dapagliflozin results in more efficient allocation of health care resources for the treatment of T2DM, and may aid patients, physicians, and payers in the selection of appropriate AHAs for the management of T2DM.

## Electronic supplementary material

Below is the link to the electronic supplementary material.

Supplementary material 1 (PDF 312 kb)
