# Supplementary material for: Cost-Effectiveness Analysis of Canagliflozin 300 mg Versus Dapagliflozin 10 mg Added to Metformin in Patients with Type 2 Diabetes in the United States
Source: Diabetes Ther. 2018 Feb 6;9(2):565–81. doi: 10.1007/s13300-018-0371-y (PMC6104269; doi:10.1007/s13300-018-0371-y)

SUPPLEMENTARY MATERIALS

Supplementary Table S1. Key Patient Baseline Characteristics[a]

Parameter	Pooled dual therapy canagliflozin studies[b]	Real-world patient characteristics
Demographics		
Age, y	55.7 ± 9.3	56.5 ± 11.8
Male, %	49.8	51.8
Duration of T2DM, y (range)	0.0-13.5	0.0-13.5[c]
Biomarkers		
HbA1c, %	7.9 ± 0.8	7.4 ± 1.6
SBP, mmHg	129.1 ± 13.1	129.1 ± 13.1[c]
BMI, kg/m^2	31.4 ± 5.8	31.4 ± 5.8[c]
Total cholesterol, mmol/L (mg/dL)	4.8 ± 1.1 (187.0 ± 40.6)	4.9 ± 1.2 (189.6 ± 47.7)
LDL-C, mmol/L (mg/dL)	2.7 ± 0.9 (105.1 ± 34.6)	2.7 ± 0.9 (104.0 ± 34.7)
HDL-C, mmol/L (mg/dL)	1.2 ± 0.3 (46.2 ± 11.7)	1.2 ± 0.3 (45.2 ± 11.7)
Triglycerides, mmol/L (mg/dL)	2.1 ± 1.5 (183.4 ± 134.0)	2.3 ± 1.6 (202.4 ± 145.8)
eGFR, mL/min/1.73 m^2	89.4 ± 18.6	94.2 ± 20.6

BMI, body mass index; eGFR, estimated glomerular filtration rate; HDL-C, high-density lipoprotein cholesterol; LDL-C, low-density lipoprotein cholesterol; SBP, systolic blood pressure; SD, standard deviation; T2DM, type 2 diabetes mellitus.

[a]Data are mean ± SD unless otherwise indicated.

[b]Data sourced from a pooled analysis of the 52-week, head-to-head study of canagliflozin 100 and 300 mg versus sitagliptin 100 mg [51] and the 52-week, head-to-head study of canagliflozin 100 and 300 mg versus glimepiride [52].

[c]Data not available in database, so assumed to be the same as the pooled analysis of canagliflozin studies.

Supplementary Table S2. Event-year and State Costs and QALY Utility Weight Inputs

Parameter	Event-Year Cost, $	State, Annualized Cost, $	Utility decrement
Patient characteristics			
Age (per 10 y)	NA	NA	–0.0235 [38]
Female	NA	NA	–0.0930 [38]
Duration of T2DM (per 10 y)	NA	NA	–0.0163 [38]
Excess body weight (per kg/m^2 over 25 kg/m^2)	NA	NA	–0.0061 [38]
Macrovascular complications			
MI	67,503.80	2,277.03	–0.028 [38]
IHD	25,599.90	2,277.03	–0.028 [38]
CHF	28,412.71	2,277.03	–0.028 [38]
Stroke	50,371.03	18,584.62	–0.115 [38]
PVD	150.69	150.69	–0.061 [38]
Microvascular complications			
Blindness	3,422.73	3,422.73	–0.057 [38]
No nephropathy			
eGFR 60-89 mL/min/1.73 m^2	0	6,404.83	0
eGFR 30-59 mL/min/1.73 m^2	0	8,531.41	–0.050 [39]
eGFR 15-29 mL/min/1.73 m^2	0	21,856.11	–0.070 [39]
eGFR <15 mL/min/1.73 m^2	0	21,856.11	–0.200 [39]
Microalbuminuria			
eGFR >90 mL/min/1.73 m^2	94.48	94.48	0

eGFR 60-89 mL/min/1.73 m^2	94.48	6,499.31	0
eGFR 30-59 mL/min/1.73 m^2	94.48	8,625.89	0
eGFR <30 mL/min/1.73 m^2	94.48	21,950.59	0
Macroalbuminuria			
eGFR >90 mL/min/1.73 m^2	130.36	130.36	–0.048 [38]
eGFR 60-89 mL/min/1.73 m^2	130.36	6,535.19	–0.048 [38]
eGFR 30-59 mL/min/1.73 m^2	130.36	8,661.77	–0.048 [38]
eGFR <30 mL/min/1.73 m^2	130.36	21,986.47	–0.048 [38]
ESRD, eGFR <15 mL/min/1.73 m^2	0	85,764.33	0[a]
Symptomatic neuropathy	1,050.02	1,316.71	–0.084 [38]
Diabetic foot ulcer	2,567.64	987.67	–0.170 [38]
Lower extremity amputation	10,812.33	2,064.12	–0.272 [38]
Hypoglycemia			
Non-severe symptomatic	0	NA	–0.0035 [59]
Severe	636.06	NA	–0.0118 [59]
AEs			
Genital mycotic infection (male)	132.74	NA	–0.0046 [60]
Genital mycotic infection (female)	132.74	NA	–0.0046 [60]
Lower UTI	125.57	NA	–0.00123 [60]
Upper UTI	136.34	NA	–0.00729 [60]
Volume depletion–related AEs[b]	83.71	NA	–0.005 [46]
Osmotic diuresis–related AEs[c]	88.50	NA	–0.005 [47]

AE, adverse event; CHF, congestive heart failure; eGFR, estimated glomerular filtration rate; ESRD, end-stage renal disease; IHD, ischemic heart disease; MI, myocardial infarction; PVD,

peripheral vascular disease; QALY, quality-adjusted life-year; T2DM, type 2 diabetes mellitus; UTI, urinary tract infection.

[a]To avoid double counting as the disutility for ESRD is included in eGFR <15 mL/min/1.73 m^2 and no kidney damage.

[b]Utility decrement assumed to be equal to that for symptomatic hypoglycemia [46].

[c]Utility decrement assumed to be similar to that of overactive bladder [47].

Supplementary Table S3. Sources of QALY Disutility in the Base Case

**Health state**	**Canagliflozin 300 mg**	**Dapagliflozin 10 mg**	**Difference**
Micro- and macrovascular complications			
MI	0.031	0.031	0.000
IHD	0.030	0.031	0.000
CHF	0.013	0.013	0.000
Stroke	0.031	0.031	–0.001
Retinopathy	0.018	0.018	0.000
CKD	0.123	0.122	0.000
Neuropathy	0.349	0.350	–0.001
AEs			
Hypoglycemia	0.184	0.222	–0.038
Other AEs	0.006	0.007	–0.001
Excess weight	0.612	0.631	–0.019
Survival[a]			–0.018
**Total[b]**			**–0.079**

AE, adverse event; CHF, congestive heart failure; CKD, chronic kidney disease; IHD, ischemic heart disease; MI, myocardial infarction; QALY, quality-adjusted life-year.

[a]For consistency with the other disutility measures, only the disutility associated with survival differences is reported (with the value of one intervention naturalized to 0).

[b]Note: This is measured as the sum of the incremental disutilities and equals the QALY differences reported earlier.

Supplementary Figure S1. Biomarker evolution curves for (A) HbA1c, (B) SBP, and (C) BMI over 30 years.

A.

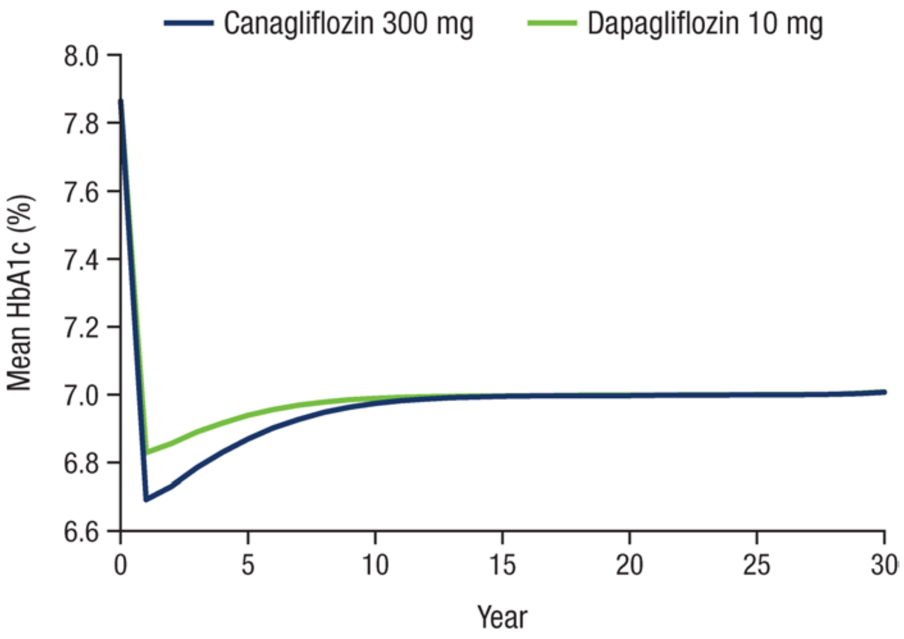

B.

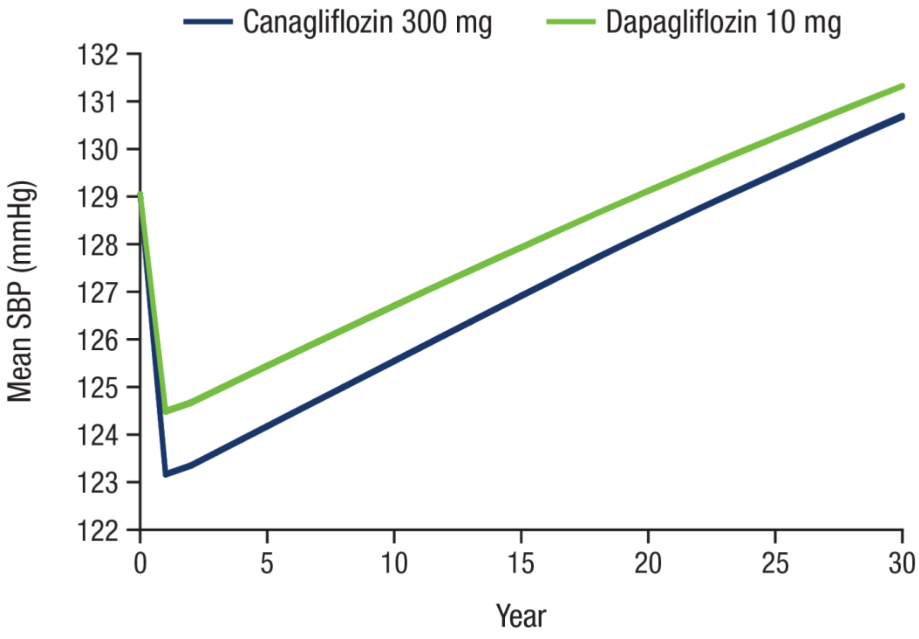

C.

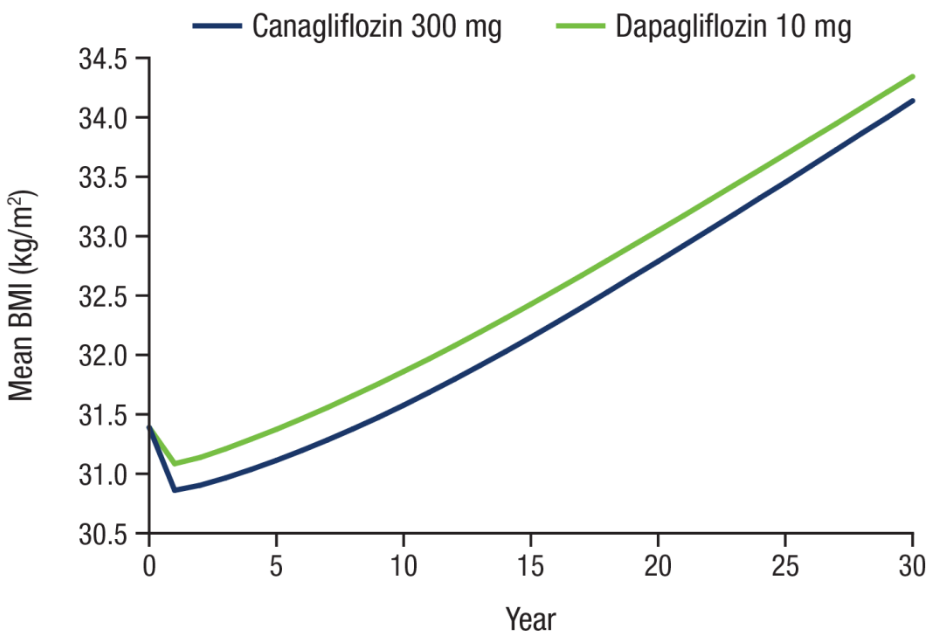

BMI, body mass index; SBP, systolic blood pressure.

Supplementary Figure S2. Simulated insulin use with canagliflozin 300 mg versus dapagliflozin 10 mg over 30 years.

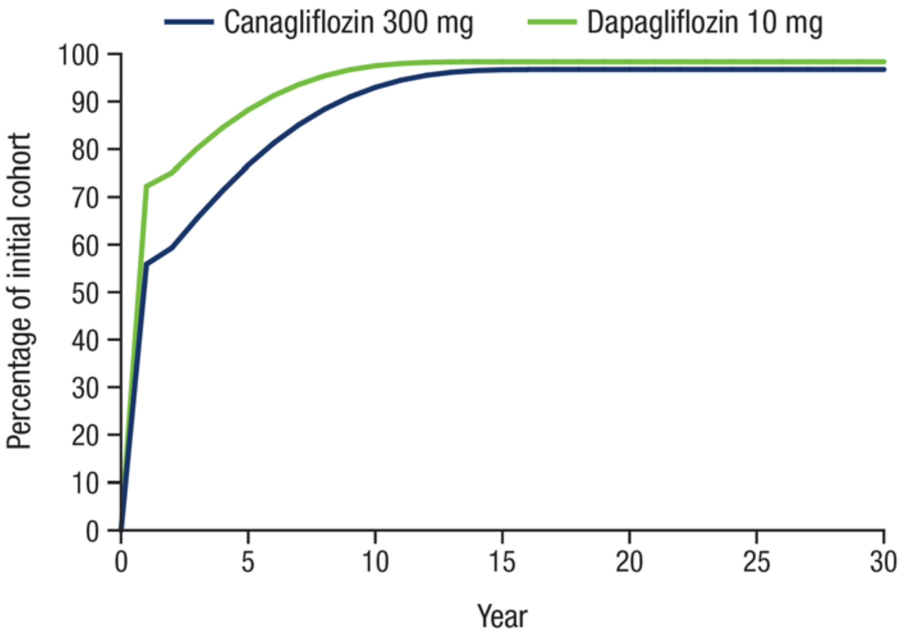

Supplement: Supplementary file 1 — Supplementary material 1 (PDF 312 kb) [file 13300_2018_371_MOESM1_ESM.pdf]
